# Quality of Life and Psychosocial Impacts of the Different Restrictive Measures during One Year into the COVID-19 Pandemic on Patients with Cancer in Italy: An Ecological Study †

**DOI:** 10.3390/ijerph18137161

**Published:** 2021-07-04

**Authors:** Maria Ferrara, Elisa Langiano, Lavinia Falese, Antonella De Marco, Elisabetta De Vito

**Affiliations:** Health Education Observatory of the Hygiene Laboratory, Department of Human Sciences, Society and Health, University of Cassino and Southern Lazio, 03043 Cassino, Italy; m.ferrara@unicas.it (M.F.); langiano@unicas.it (E.L.); liberopietrantuono2003@gmail.com (A.D.M.); devito@unicas.it (E.D.V.)

**Keywords:** cancer, containment, coronavirus, COVID-19, lockdown, mental health, pandemic, quality of life

## Abstract

Background: The aim of the study was to assess the perceived quality of life and the psychosocial impact of the various restrictive measures due to COVID-19 pandemic on cancer patients in Italy, as well as their perception of the relationship with doctors and caregivers. Methods: This study compares three population-based observational studies of patients with cancer carried out in three consecutive time periods characterized by different restrictive measures using a self-administered online questionnaire. Results: Among the basic needs, psychological and medical support appeared to be prevalent; so did the need for safe transportation to reach the treatment facilities. Internet was the main source of information on the coronavirus. Although 74.6% of the total number of patients did not give up hospital therapies, 34.8% complained about variations in the continuity of treatment, with different percentages in the three samples. The majority of the sample (73.8%) was worried of being infected, but 21.9% did not share their anxieties and worries with others. The multivariate regression analysis showed that a pessimistic perception of quality of life was influenced by living in extra-urban areas and alone (OR = 1.4; OR = 2.1); while a perception of a reduced physical function result affected by the state of anxiety and stress (OR = 1.9) and the difficulties in continuity of medical assistance (OR = 2.2). The scoring of the SF-12 in the Physical Component Summary and Mental Component Summary scores showed a fluctuating trend throughout the three periods investigated. Conclusions: It is important for health professionals, caregivers and social workers to identify the new needs in order to enhance home care interventions, personalize and optimize care, ensure continuity of care and guarantee a high quality of life even in a health emergency situation.

## 1. Introduction

The COVID-19 pandemic had a different timeline of spread around the world, and countries decided to adopt lockdowns, quarantines and restrictive measures with a lot of variability on the implementation of the set regulation [1].

Given the high level of contagiousness, almost every country has adopted restrictive measures of social distancing and home isolation [2].

The Italian government has implemented different containment measures such as social distancing, cases isolation, quarantine, lockdown and contact tracing according to the infected population and number of deaths [3]. These measures were at first limited to the north of Italy only (starting from February 2020), then subsequently extended to the rest of the country on the 8th of March 2020, and implemented according to the current status of the so-called waves in relation to the number of positive cases, hospitalizations and deaths.

There is no doubt that the COVID-19 disease and the restrictive government measures are harming the mental health of individuals around the world, causing fear, frustration, anger and a long list of complex negative emotions [4,5].

The pandemic represents a stressful event that affects the population at both individual and general levels. A paper published during the outbreak of the pandemic in China reported that individuals were affected by high levels of fear, panic and anxiety, and hypothesized that these feelings may have also had implications for other health measures [6]. At the population level, the pandemic caused several difficulties such as unemployment and reduced income, losses of family and friends, education continuity, problems in medical assistance, domestic violence and poor distribution of basic needs [7]. All these multifaceted adversities, together with fear for the future and fear of contracting the virus and dying, are sources of elevated psychosocial stress that deserve in-depth analysis [8,9].

The decrees, regulations and protocols that were issued during the pandemic period aimed mostly at protecting that part of the population considered most vulnerable, especially from a physical health point of view, and took less into consideration the psychological impact, which may require specific tailor made interventions.

While most of the clinical and research efforts focused on reducing the consequences of the COVID-19 disease on the physical health [10], its impact on mental health, especially of specific population groups such as the ones living with chronic conditions, have been little investigated [11].

Recent studies in China highlighted that the COVID-19 pandemic has caused difficulties to individuals who need regular assessments and treatments at hospitals, such as cancer patients, especially due to the unavailability of healthcare resources engaged in virus-related activities [12].

Cancer is the second leading cause of death globally and, according to the Italian National Institute of Statistics (ISTAT), it was responsible for an estimated 180,000 deaths among the approximately 600,000 deaths that occurred in 2016 in Italy [13,14].

In 2019, there were about a thousand new cases of cancer per day in Italy with a prediction for the year 2020 of about 371,000 new cases of malignant tumors. The most common causes of cancer death in 2019 were cancers of breast (53,500 new cases), colorectal (49,000), lung (42,500), prostate (37,000) and bladder (29,700) [14].

In the last years, however, mortality from cancer has been decreasing in both sexes, and overall recovery rates have improved, thanks especially to the greater adherence to screening programs, which has allowed for early detection of neoplasm and greater effectiveness of therapies [15]. On the other hand, the decrease in early diagnoses during the pandemic predicts an increase in late diagnoses, and consequently greater difficulty in recovery [16,17].

As for other chronic degenerative diseases, the onset, course and outcomes of cancer are associated with social factors and lifestyles, but also psychological factors.

Cancer diagnosis brings high levels of psychological stress to patients as well as changes in patients’ life, activities and relationships [18,19]. Psychological factors can be considered risk factors for specific types of cancer and could also aggravate the course of the disease [20].

During the COVID-19 pandemic, for cancer patients, the severity of the virus, all the implemented restrictive measures and the way medical and surgical care were delivered, have been a source of concern and anxiety due to the perception of higher mortality risk [21], the fear of contracting the virus (80% of cancer patients concerned according to a study conducted in Denmark) [22], fear of increased risk of complications if infected, fear of no longer receiving the necessary cancer treatment and fear of being far away from family and friends due to social isolation [21,23,24]. According to a recent research about the impact of the COVID-19 pandemic on patients with cancer with data collected through social media, the major concerns relate to delayed diagnosis or treatments and increased risks due to the lowered immune defenses [25]. Being concerned about contracting the virus was also found to be associated with lower quality of life in cancer patients [20], especially when living alone [26].

Quality of life (QOL) has been defined by the World Health Organization “as an individual’s perception of their position in life in the context of the culture and value systems in which they live and in relation to their goals, expectations, standards and concerns” [27]. A more specific explanation of the concept of QOL for cancer patients was given by Ferrell and Dow and includes the concept of physical well-being, psychological well-being, social well-being and spiritual well-being [28]. Quality of life perception is very subjective and differs among individuals even when they share the same health status [29].

Risk factors of anxiety and low quality of life for patients with cancer during COVID-19 include specific socio-demographic characteristics such as gender (being female) and age and lifestyle (e.g., family environment) [30].

Individuals who had to undergo oncological screening but postponed it for fear of contagion, individuals with delayed treatments because of the emergency, thus delaying diagnosis, as well as those patients struggling with complex pathological situations, all experienced strong psychological and emotional distress regardless of the severity of their disease [31].

In cancer patients, in fact, increased risk of mortality and difficulties in accessing health facilities to undergo medical examinations, tests and treatment for fear of becoming infected is intensifying the risk of developing mental disorders or worsening existing problems [32]. In addition, mass quarantine and expanded restrictions on public transport represent a major barrier to access treatment and support facilities.

When analyzing the impact of COVID-19 on cancer patients, it must be taken into consideration that these individuals may have different personalities and beliefs and not all of them have faced the emergency at the same stage of their cancer illness.

The patients, who were diagnosed with cancer a long time before the start of the pandemic, reactivate their fears, perception of danger, sense of threat of death and a state of alarm that they first felt when they were diagnosed with their disease.

The patients undergoing chemotherapy or radiotherapy during the pandemic, on the other hand, have already entered a process that helps them to regain a sense of control, the so-called adaptation phase. In this phase, they are able to handle a good amount of stress and to apply mechanisms of resilience, i.e., adaptation to the situation.

For those at the beginning of their cancer journey, a diagnosis during the pandemic or right before brings two types of traumas: one related to the discovery of the illness and another one related to the pandemic itself and its consequences.

Ultimately, patients who were newly diagnosed and admitted for surgery to remove tumors during the pandemic had a very different experience from what they would have had before the outbreak. The hospital became a building with restricted access in order to protect patients, visitors and medical staff, and this triggers an overwhelming sense of loneliness [33]. Although medical teams can provide the patient with great assistance and care to, they cannot replace the family. In our perception—supported by scientific literature—we hypothesize that the quality of life and the social impact of COVID-19 pandemic on cancer patients change accordingly to the restrictive measures adopted in their country.

The aim of this research is, therefore, to assess the quality of life and the psychosocial impact of the COVID-19 pandemic as well as the perception of changes in the relationship with doctors and caregivers of cancer patients in Italy, and to identify possible risks and protective factors for mental health outcomes. A specific aim of our investigation is to assess whether these variables change according to the different time frames and restrictions applied in the country.

In particular, we expect to find, in the data collected during the summer period (when there were fewer restrictions due to fewer cases), better quality of life and lower psychosocial impact.

The results of our study can be useful to provide the necessary elements to elaborate socio-assistance interventions aimed at maintaining or improving the quality of life and the caretaking of chronicity of cancer patients on the basis of their physical and also psychological needs.

## 2. Materials and Methods

No aprioristic statistical calculation of the sample size and non-probability random sampling was performed. Cancer patients undergoing medical treatment of both sexes were invited to participate in the survey. We used an online social media strategy to recruit survey participants, and we collected data through an anonymous online questionnaire.

The questionnaire was uploaded on the Google Form platform and invitations were sent on the social networks Facebook (Facebook Inc., Menlo Park, CA, USA) and WhatsApp (Facebook Inc., Menlo Park, CA, USA). Patients interested in participating in our research were invited to access the link to the questionnaire and to read the detailed information on the aims of the study and the statement about anonymous mode. Informed consent and authorization to process sensitive data was also requested in accordance with Italian law (196/2003 and subsequent amendments and additions concerning personal data). Both authorizations were mandatory fields to continue the survey.

This study compares three population-based observational studies of oncological patients undergoing treatment during the COVID-19 pandemic, performed in three different time periods (T0–T2) characterized by different restrictive measures adopted by the Italian national and regional government. The first collection of data was done from April to June 2020 (T0), the second from July to September 2020 (T1) and the third from November 2020 to January 2021 (T2). These three time periods are subsequent to the issuing of the first Italian Prime Ministerial Decree (9 March 2020) released in order to contain and control the spread of the COVID-19 virus throughout the country and correspond to different restrictive measures with T1 having less stringent restrictions.

This method made it possible to promptly collect the perceptions of the chosen target audience, who reported their impressions and feelings while experiencing them.

The study was approved by the Institutional Review Board of the University of Cassino and Southern Lazio.

### 2.1. Survey Tool

We used an easy to understand and to fill in online self-administered questionnaire built ad hoc, in Italian, by the Health Education Observatory of the Hygiene Laboratory of the Department of Human Sciences, Society and Health of the University of Cassino and Southern Lazio.

The questionnaire consists of 42 questions. The first part of the questionnaire (items 1–8) aims to collect demographic information (gender, age, area of residence, level of education, occupation, etc.) referred to categories defined by ISTAT [14]. The second part (items 9–12) examines health status (type of cancer, comorbidities, type of therapy, etc.) as defined by the Italian Manual ICD9CM 2007 [34]. We developed the part of the questionnaire with cancer-specific questions after carefully consulting the literature review using the following keywords: Cancer disease, Cancer disease AND gender, Cancer disease AND geographic origin, Cancer disease AND family environment characteristics Cancer disease AND lifestyle, Cancer disease AND quality of life.

The third section of the questionnaire (items 13–30) seeks information on family environment, lifestyle, personal needs, etc.

The fourth and final section measures the perception of quality of life, more specifically physical and mental health, through the Short Form-12 (SF-12) instrument, validated on the Italian population by Kodraliu et al. (2001) [35]. This is a short form of the more extended Short Form-36 items Health Survey, more commonly known as SF-36, which allows us to describe the health status of a group of individuals by investigating only two synthetic indexes: PCS (Physical Component Summary), index of physical status and MCS (Mental Component Summary) index of mental status [35]. This tool helped us to analyze patients’ needs through the assessment of general distress levels and the presence of symptoms of depression, anxiety and stress related to the coronavirus epidemic. The synthesis of the scores, obtained by comparison with normative values, provides a snapshot of the health status of the population under study. The general population has a score of 50 ± 10 SD. Scores below 43 indicate severe limitations in the quality of life of the patients [36]. The strengths of this questionnaire, which were decisive for the choice of the tools used in this study, are its brevity and ease of use.

### 2.2. Statistical Analysis

In order to analyze the data from the questionnaire, a descriptive univariate analysis was first carried out to represent synthetically our data set and to describe the socio-demographic and clinical characteristics of the total sample and of the three different samples by means of a simple frequency distribution.

A bivariate analysis was performed to investigate the association between certain socio-demographic factors (gender, level of education and occupation) and the MCS and PCS domains of the SF-12. The respondents’ places of residence were recoded using a binary variable (yes/no) named ‘Urban area’. This variable was included in the regression model in order to assess their difficulties in reaching health care provider services. We hypothesized that individuals living in the suburbs or small areas would have more difficulty in reaching hospitals or health facilities, especially if they are used to riding on public transportation, compared to those living in urban areas.

The results obtained from the SF-12 questionnaire were reported as a numerical score, which was standardized and ranked on a scale from 0 to 100, with higher scores indicating better self-reported health status. Two summary scores were reported from the SF-12: the MCS and the PCS. PCS and MCS were categorized as dichotomous variables considering values above and below the mean, respectively. In order to assess the factors associated with the severity of depressive symptoms, anxiety and stress by SF-12, multivariate linear regression models were performed. Logistic regression was used to study, in the total sample (n. 769) the relationships between independent variables such as having a pre-existing mental disorder (suffering from anxiety or depression) and being a woman and more specific variables such as subjective perception of health status, family network and geographical area.

The logistic regression tested the influence of some socio-demographic factors as independent variables on MCS and PCS, as dependent variables. We created dummies for gender (“1” woman and “0” man), age (“1” ≤ 40 and “0” ≥ 41), family network (“1” living alone and “0” living with family), geographical area (“1” urban area and “0” suburban area), and date of last treatment by type of cancer (“1” ≤ one year and “0” > ≥ one year). Considering the aims of the study, this analysis has been performed to the total sample and to the samples at T0–T2.

The independent variables were: having a pre-existing mental illness disorder, being female. The logistic regression models were adjusted for several socio-demographic characteristics, such as gender, employment status, physical and/or psychological comorbidity, subjective perception of health status, family network, level of life satisfaction and continuity of care. Multiple logistic regression was performed using two different models to test the influence on MCS and PCS of socio-demographic variables, lifestyle, social network, geographic area and use of continuity of care as independent variables.

Statistical analyses were performed using the EpiInfo 3.5 statistical package (Centers for Disease Control and Prevention, Atlanta, GA, USA), the level of statistical significance was set at *p* < 0.05.

## 3. Results

The total sample consisted of 769 participants (T0 261, T1 218, T2 290), mainly females (85.3%), with an average age of 42.7 years ± 15.5 SD. Slightly more than half of the sample (51.9%) had a high school diploma and 33.1% a university degree. More than half of the subjects had a stable relationship and lived with their partner (53.2%), while few lived alone (7.9%). The sample came from all over Italy and mainly from a suburban area (76%). About 20% of the participants were employed and were going to work regularly as before the pandemic. Additionally, 17.2% of the sample was on sick leave, 18.1% retired, 14% consisted in housewives, 5.6% were on lay-off and 18.6% switched to smart working from home during the pandemic. No one reported being in isolation because they were COVID-19 positive, although 4.5% of the sample stated that they were in precautionary isolation (Table 1).

The most common forms of cancer are breast cancer (28.3%), followed by colorectal cancer (14.4%), leukemia (11.3%), lymphoma (9.8%), lung cancer (5.8%) and ovarian cancer (4%).

Some of the respondents had acquaintances/friends/family members who were infected and were quarantined at home, who were hospitalized or who died due to COVID-19 (23%, 14.9% and 13.1%, respectively). About 80% spent more time on the internet than usual, most frequently for instant messaging (84%). The internet resulted as the main source of information about the coronavirus for most of them (79.8%), while doctors (specialists or general practitioners) were little mentioned (17.1%). Few patients had a pre-existing physical illness (11.1%), and a small percentage (5.9%) reported pre-existing psychological distress, most frequently anxiety (42.2%) and depressive disorders (36.1%). During the pandemic, almost half of the respondents perceived their health to be good or fair (47.9% and 40.3%, respectively), while a small percentage (10.9%) considered it very poor. The reported primary need was psychological support (59.4%), followed by medical support (38.5%).

Using safe transportation to reach hospital facilities for treatment was also one of the basic needs of the sample (30.1%). Although 74.6% of the total number of patients, with differences in the three moments taken into consideration (65.1% T0, 79.1% T1, 69.4% T2) did not give up hospital treatment, 34.8% complained about variations in the continuity of treatment, with different percentages at T0 (42.1%), T1 (36.2%), and T2 (40.1%). Most of the sample (75.2%) were worried about being infected, but 26.8% of them did not share their anxieties and worries with others.

The scores obtained from the items of the SF-12 questionnaire made it possible to plot the physical health (PCS) and the mental health (MCS) summary scales of the total sample that reported a mean score of 51.4 ± 6.2 SD and 50.1 ± 7.2 SD, respectively.

Table 2 shows the elaboration of PCS and MCS. Statistically significant data (with 95% confidence interval) emerged with lower scores in both scales in relation to female gender (PCS male 49.2 ± 7.4 SD; MSC male 47.8 ± 8.2 SD; *p* < 0. 05), to educational qualification (PCS 49.9 ± 7.3 SD; MCS 46.4 ± 8.2 SD; *p* < 0.01) and in relation to employment level (PCS 50.8 ± 6.2SD; MCS 49.7 ± 7.1SD; *p* < 0.05).

By calculating the average score in the three groups of responders recruited at the three time points considered, it emerges that the Physical Component Summary and Mental Component Summary scores show a fluctuating trend with low overall values at T0 (PCS 47.7 ± 9.2 SD; MCS 46.1 ± 8.3 SD), a slight increase at T1 (PCS 49.7 ± 7.9 SD; MCS 48.3 ± 5.4 SD) and a new decrease at T2 (PCS 48.1 ± 6.9 SD; MCS 47.7 ± 4.7 SD) (Table 3).

Linear regression models showed a significant correlation between the presence of pre-existing psychological distress and an increase in the severity of depressive-anxiety symptoms (MCS) due to the pandemic *r* = 0.3; (*p* < 0.05).

Multivariate regression analysis showed that a pessimistic perception of quality of life was influenced by living in suburban areas (OR = 1.4; 95% CI: 1.09–3.1; *p* < 0.05) and alone (OR 2.1; 95% CI: 2.09–4.3; *p* <0.05); while perception of reduced physical function was influenced by anxiety and stress (OR = 1.9; CI 95%: 1.4–3.01; *p* < 0.05) and difficulties in continuity of medical care (OR = 2.2; CI 95%: 1.1–4.8; *p* < 0.05). Multivariate regression models, applied to the whole sample, adjusted for the period of exposure to the pandemic and related restrictive measures were significantly associated with worse depressive symptoms (OR = 1.9; 95% CI: 1.3–3.2; *p* < 0.05) and stress (OR = 2.1; 95% CI: 1.7–3.5; *p* < 0.05). The risk of major depressive symptoms, anxiety and stress was higher in females (OR = 2.5; 95% CI: 1.6–3.9; *p*< 0.05) and in people with pre-existing psychological distress (OR = 3.1; 95% CI: 2.4–4.6; *p* < 0.05). In addition, we found that subjects who showed higher levels of satisfaction with their lives and with a stable family network reported less psychosocial impact of the pandemic (OR = 0.3, 95% CI: 0.09–0.8; *p* < 0.05 and OR = 0.4, 95% CI: 0.1–0.9; *p* < 0.05, respectively).

A specific regression model, applied in the three groups considered, was used to test any changes in the relationship among a group of identified explanatory variables (sex, educational qualification occupation, geographical area of origin, discontinuity in therapeutic care) and physical and mental health. Statistical significance was found on physical and mental health scores below the national mean values.

For MCS (Table 4), women, those living alone and in suburban areas were significantly less likely to have MCS above the national mean value.

For PCS (Table 5), lower SF-12 scores are associated with: age, gender, and type of cancer.

## 4. Discussion

The present study suggests that the pandemic we have been experiencing represents an unexpected and traumatic event that has a negative impact on the lives and mental health of the general population, especially those affected by chronic conditions.

The findings of our research also suggest that levels of anxiety, depression and stress change over time, being more elevated in the first weeks of the pandemic, as confirmed in our regression model adjusted for socio-demographic characteristics of the respondents. This would confirm that the duration and harshness of the restraint significantly affect not only physical health, but psychological and social health as well. In the present investigation, the overall health status of the sample examined, measured by the synthetic indices of physical state (PCS) and psychological state (MCS), was slightly higher than the national average, probably due to the sampling mode. According to the Italian National Institute of Statistic (ISTAT), the average national physical state score (PCS) is 50.7 (vs. 51.7 in our sample), while the average mental state score (MCS) is 48.9 (vs. 50.4 in our sample) [37]. However, the analysis of the average index of PCS and MCS in three samples considered at three different moments of data collection shows a fluctuation of such scores. The average scores resulted lower than the national average values at T0 and T2, the two periods in which the measures adopted had greater restrictions, and values similar to the national average at T1, which indicates an improvement in self-reported health status in relation to the relaxation of restrictive measures following the improvement in the epidemiological data of the pandemic. The self-reported mental health status was slightly worse when compared to the physical health status, and differences between periods were more pronounced. Physical and mental health affect each other but, whereas physical symptoms are more often the focus of health providers, mental and social consequences of cancer disease are less emphasized and recognized [38].

Although research about the assessment of the quality of life at different stages of the disease in scientific literature can be found [38,39], as far as we know, there are only a few studies investigating the influence of the continuity of treatment due to a world pandemic on physical and mental health. In our investigation, the continuity of treatment during the COVID-19 pandemic was a matter for concern for many patients (34.8%) and the percentage of patients complaining differs according to the restrictive measures put in place (less complain at T1), as we had hypothesized, and is consistent with the findings of Islam et al. (2021) [21].

In our study, females were found to be at higher risk of developing depressive anxiety symptoms, as already shown in a previous study in a small Italian population sample [40], in a recent study in the United States [21] and in previous outbreaks. This result may be due to a higher incidence of anxiety-depressive disorders in women [41] and depressive status and mood swings in women [42], and also in community samples [43].

The results of linear regression and multivariate regression analysis for the overall sample are consistent with the findings of previous studies that identified as risk factors for a low perception of quality of life of cancer’s patients some demographic variables (i.e., age, gender, marital status, education, area of residence) and medical variables (i.e., treatment variables) [21,28,44]. A recent study about quality of life of patients with thyroid cancer during COVID-19 confirmed that women and younger patients have higher concerns about their quality of life over the pandemic outbreak, but it did not find any differences across different clinical status groups [31].

Moreover, having pre-existing mental health problems is a significant risk factor for the development of depressive symptoms, anxiety and stress [5].

These findings suggest the need to provide appropriate and tailored support interventions as early as possible to cancer patients with symptoms of psychological and emotional disorders, a vulnerable segment of the population that were neglected during the initial stages of the pandemic [45,46].

The participants in our survey reported an increase in time spent on the Internet, probably seeking information and emotional support, which we found directly linked to the risk of developing mental health complications, confirming the findings of a qualitative study on the use of social media during the lockdown in patients with cancer [25]. The hypothesis of a protective effect played by the Internet on mental health was therefore not confirmed in our study maybe because of the diffusion through the Internet of unreliable information and fake news, which might have increased the levels of anxiety and depressive symptoms in lonely people with lower levels of education [47].

In a situation of health and social risk, communication must be done accurately and there is need to receive adequate training for media professionals in order to provide impartial and realistic information during catastrophic events.

In our study, being unemployed, retired or housebound was significantly associated with higher levels of anxiety-depressive symptoms. Our results are in line with another study carried out in the United Kingdom that showed that, belonging to a socio-economically disadvantaged group, increased gradually the risk of developing problems of psychosocial distress during the first three weeks of the lockdown [48]. It is therefore important to undertake comprehensive, multi-level socio-economic initiatives aimed at reducing the negative effects of the pandemic on society.

Finally, good levels of family support were reported by the sample that participated in our survey. This may be due to the fact that the Italian socio-cultural context, with strong family ties and social relationships, may have had a positive impact on the perception of mutual social support [49] that is very important to face the challenges of the disease [39].

### Strengths and Limitations

Our research was conducted across the Italian peninsula with a fair sample of the Italian cancer population. The data collection lasted almost 10 months, and this allowed us to compare three similar samples at different moments of the pandemic. Validated and reliable assessment tools were used to investigate different domains of health and perception of quality of life level. The recruitment strategy helped us to collect a fair number of responses well distributed all over the Italian peninsula; however, the sample lacks statistical representativeness due to the sampling procedure, the unique population group and the choice of the instrument (online recruitment). We are aware that using an online tool can be considered a limitation to the research as it may have excluded older patients, those living in socially disadvantaged settings and those not using social media. However, this choice allowed us to reach, in short time and during a pandemic situation, a specific target of the Italian population and it may be a first way of obtaining information during emergency times that should be further explored in the future. Moreover, we acknowledge that our results are related to depressive or anxious symptoms and not necessarily to diagnosing of depressive/anxious disorders.

One last limitation to the study is that participants do not coincide at T0, T1 and T2. Having the same sample and a research design based on a longitudinal investigation, would have strengthened the statistical significance of the findings.

## 5. Conclusions

Although physical isolation and lockdown are essential public health measures to contain the spread of the COVID-19 pandemic, they represent a serious threat to the psychological and social health and well-being of the general population especially those affected by health problems.

The emotional, social and relational difficulties that have emerged require strong resilience. It is important for health professionals, caregivers and social workers to identify new needs in order to enhance home care interventions, personalize and optimize care, ensure continuity of care and guarantee a high quality of life even in a health emergency situation.

In addressing measures that can be put in place to deal with the psychosocial impact of the pandemic, a first distinction must be made among interventions during the crisis and interventions after the crisis. The second distinction that needs to be made is among those whom were quarantined because they were infected or were in contact with infected people, and those whom only underwent lockdown.

Some practical implications of this research include: promoting mass screening campaigns in order to identify the presence or the risk to develop mental disorders; the spreading of recommendations about how to deal with the mental health consequences of the pandemic and the development of tailored innovative psychosocial interventions in order to help the population at risk. Moreover, health providers should offer equitable access to digital health tools and platforms, as well as implement local territorial medicine services in order to guarantee in-person support (home care) and continuity of treatments [25,50].

A multi-disciplinary approach involving oncologists, family doctors, social workers, psychologists and, in some cases, psychiatrists, should be pursued.

During the pandemic, the main mental treatment ought to be aimed at counteracting fear. Meditation techniques, mindfulness and psychological support, as well as online counselling, can be very helpful during this time.

After the critical phase, attention will have to be focused on ensuring well-being at work and monitoring over time [51].

Therefore, investing in mental health services and programs at a national level, which have suffered from limited funding for years, is now more important than ever.

## Figures and Tables

**Table 1:** Study population.

Variable	Total	95% CI	T0	95% CI	T1	95% CI	T2	95% CI
Sample	n. 769		n. 261		n. 218		n. 290	
Age (mean)	42.7 years ± 15.5 SD	----------	41.2 years ± 13.2 SD	---------	44.3 years ± 12.5 SD	---------	43.9 years ± 12.9 SD	----------
Gender								
male	85.3	83.1–88.2	76.2	73.2–79.1	79.2	75.3–82.8	81.0	76.5–84.6
female	14.7	11.8–17.9	23.8	21.4–25.9	20.8	17.8–23.1	19.0	16.2–22.9
Educational level								
no formal education	0.6	0.3–0.8	0.4	0.1–0.6	0.7	0.3–0.9	0.5	0.2–0.7
primary	14.4	10.9–16.9	10.1	7.9–13.1	13.1	10.4–16.4	11.4	10.6–12.7
secondary	33.1	29.2–36.1	36.0	31.7–39.6	37.2	33.3–40.1	36.4	32.2–39.9
university	51.9	46.2–56.7	53.5	47.4–58.7	49.0	44.4–53.1	51.7	50.0–53.1
Employment situation								
continues to work regularly	20.0	19.1–21.9	24.1	20.9–26.1	19.6	18.7–22.8	19.9	18.5–22.1
working from home	18.6	17.1–20.2	21.9	18.1–26.2	17.4	16.3–18.6	18.1	16.8–21.4
retired	18.1	16.8–19.9	16.2	15.1–17.3	20.1	18.6–23.1	19.6	10.7–20.9
sick leave	17.2	16.1–18.1	19.3	18.2–21.3	18.5	17.3–19.9	18.0	16.4–19.7
layoffs	6.1	5.3–7.4	8.2	7.3–9.1	7.8	6.2–9.7	6.9	5.1–8.1
housewife	5.6	3.9–7.9	4.9	3.6–5.8	6.0	5.3–7.9	5.9	4.3–6.9
other	14.4	13.3–15.5	5.4	4.2–9.5	10.6	8.7–12.1	11.6	10.5–12.8
Relationship Status								
live with their partner	53.2	49.8–56.1	54.8	53.5–56.1	52.7	50.8–55.3	53.0	51.2–55.6
live with the original family	18.2	15.7–19.8	19.9	18.1–21.9	17.1	15.9–19.2	17.9	15.7–19.9
live alone	7.9	5.8–10.0	6.4	5.2–7.9	9.5	8.3–11.1	8.0	5.9–10.1
other	20.7	18.8–22.1	18.7	16.6–20.2	20.7	18.8–22.4	21.1	18.8–23.6
Area of residence								
urban area	24.0	20.2–26.8	27.1	25.4–30.1	26.2	23.1–29.7	25.9	22.2–28.6
suburban area	76.0	74.1–79.7	72.9	70.7–76.5	73.8	69.9–78.1	74.1	71.6–82.5

**Table 2:** Bivariate analysis processing of PCS and MCS.

Variable	Physical Health (PCS)	*p* *	Mental Health (MCS)	*p* *
Gender				
Male	49.2 ± 7.4 SD	0.04	47.8 ± 8.2 SD	0.05
female	47.1 ± 6.2 SD		44.2 ± 5.3 SD	
Educational level				
higher	49.9 ± 7.3 SD	0.03	46.4 ± 8.2 SD	0.02
lower	46.8 ± 5.5 SD		44.3 ± 6.3 SD	
Employment situation				
employed	50.8 ± 6.2 SD	0.03	49.7 ± 7.1 SD	0.05
not employed	48.7 ± 5.9 SD		43.5 ± 4.6 SD	

* 95% confidence interval.

**Table 3:** Average score SF12 (PCS and MCS) in the three groups of responders recruited at the three time points considered.

Physical Health (PCS)			Mental Health (MCS)		
T0 (n. 261)	T1 (n. 218)	T2 (n. 290)	T0 (n. 261)	T1 (n. 218)	T2 (n. 290)
47.7 ± 9.2 SD	49.7 ± 7.9 SD	48.1 ± 6.9 SD	46.1 ± 8.3 SD	48.3 ± 5.4 SD	47.7 ± 4.7 SD

**Table 4:** Logistic regression model relating groups of variables and MCS.

		MCS	
		OR	95% CI
**Total (n. 769)**			
Gender	male	1	
	female	1.93	1.02–3.29
Relationship Status	live with family	1	
	live alone	3.82	1.91–4.49
Area of residence	urban area	1	
	suburban area	1.78	1.09–3.01
**T0 (n. 261)**			
Gender	male	1	
	female	1.35	1.08–2.99
Relationship Status	live with family	1	
	live alone	2.61	1.04–3.19
Area of residence	urban area	1	
	suburban area	1.46	1.01–2.74
**T1 (n. 218)**			
Gender	male	1	
	female	1.14	1.09–2.25
Relationship Status	live with family	1	
	live alone	1.08	1.01–2.09
Area of residence	urban area	1	
	suburban area	1.21	1.08–2.47
**T2 (n. 290)**			
Gender	male	1	
	female	1.42	1.11–2.78
Relationship Status	live with family	1	
	live alone	1.05	1.01–2.01
Area of residence	urban area	1	
	suburban area	1.09	1.02–2.33

**Table 5:** Logistic regression model relating groups of variables and PCS.

		PCS	
		OR	95% CI
**Total (n. 769)**			
Age	≤40	1	
	≥41	1.61	1.10–2.89
Gender	female	1	
	male	0.82	0.61–0.99
Conclusion of cancer treatment for type of cancer			
breast cancer	≤one year	1.24	1.04–1.95
	>one year	1	
lymphoma	≤one year	1.88	1.23–3.02
	>one year	1	
colorectal cancer	≤one year	1.09	1.01–1.58
	>one year	1	
leukemia	≤one year	1.38	1.06–2.07
	>one year	1	
**T0 (n. 261)**			
Age	≤40	1	
	≥41	1.13	1.06–1.69
Gender	female	1	
	male	0.96	0.81–0.99
Conclusion of cancer treatment for type of cancer			
breast cancer	≤one year	1.05	1.03–1.78
	>one year	1	
lymphoma	≤one year	1.06	1.01–1.46
	>one year	1	
colorectal cancer	≤one year	1.20	1.03–2.28
	>one year	1	
leukemia	≤one year	1.31	1.06–2.01
	>one year	1	
**T1 (n. 218)**			
Age	≤40	1	
	≥41	2.01	1.33–2.73
Gender	female	1	
	male	0.71	0.62–0.89
Conclusion of cancer treatment for type of cancer			
breast cancer	≤one year	1.08	1.01–1.96
	>one year	1	
lymphoma	≤one year	2.05	1.37–3.04
	>one year	1	
colorectal cancer	≤one year	1.09	1.03–1.18
	>one year	1	
lung cancer	≤one year	1.70	1.34–2.31
	>one year	1	
**T2 (n. 290)**			
Age	≤40	1	
	≥41	1.51	1.01–2.09
Gender	female	1	
	male	0.82	0.43–0.91
Conclusion of cancer treatment for type of cancer			
breast cancer	≤one year	1.49	1.22–3.01
	>one year	1	
lymphoma	≤one year	2.34	1.92–3.38
	>one year	1	
colorectal cancer	≤one year	1.44	1.06–2.06
	>one year	1	

## Data Availability

The data presented in this study are available on request from the corresponding author.
